# Comparative Strength Development and Microstructural Characteristics of Individual Industrial Solid Waste Systems Treated with Sodium Silicate

**DOI:** 10.3390/ma19183978

**Published:** 2026-09-19

**Authors:** Zhifeng Ren, Jiajie Tong, Xiang Mao, Jianhong Fang, Long Han, Yifan Xiang

**Affiliations:** 1 College of Architecture and Energy Engineering, Wenzhou University of Technology, Wenzhou 325035, China15267539341@163.com (J.T.); 2 Zhejiang Xingchuang Environmental Protection Group Co., Ltd., Wenzhou 325035, China; 15858019578@163.com; 3 School of Civil Engineering, Sun Yat-Sen University, Zhuhai 519082, China; 4 Qinghai Research Institute of Transportation, Xining 810003, China; 13709719909@163.com; 5 Qinghai Provincial Highway Bureau, Xining 810008, China; 17609780979@163.com

**Keywords:** individual industrial solid wastes, sodium silicate, unconfined compressive strength, crystalline phase composition, microstructure

## Abstract

Bulk industrial solid wastes are promising materials for clay stabilization, but the independent performance of different waste types under identical conditions remains insufficiently understood. In this study, fly ash (FA), coal gangue (CG), and ground-granulated blast-furnace slag (GGBS) were independently incorporated into a low-liquid-limit clay matrix treated with sodium silicate. Waste dosages of 10%, 15%, and 20% and curing ages of 3, 7, and 28 days were investigated using unconfined compressive strength (UCS) tests. Representative 28-day specimens were further examined by X-ray diffraction (XRD) and scanning electron microscopy (SEM). The three systems exhibited distinct dosage- and age-dependent UCS responses, and strength did not increase monotonically with waste dosage. At 15% dosage, the 28-day UCS values of the FA, CG, and GGBS systems were 0.26, 0.26, and 0.29 MPa, respectively. XRD showed broadly similar crystalline phase assemblages, while SEM revealed differences in particle morphology and local surface deposits. The results provide a controlled comparison of the independent performance of FA, CG, and GGBS in clay stabilization under the investigated conditions.

## 1. Introduction

The large-scale generation and long-term stockpiling of industrial solid wastes have become major resource and environmental concerns. Typical industrial solid wastes, such as fly ash (FA), coal gangue (CG), and ground granulated blast furnace slag (GGBS), are produced in large quantities and show concentrated sources and pronounced regional distributions. Their long-term accumulation not only occupies land resources, but may also cause dust emissions, leachate pollution, and potential ecological risks [1,2,3,4]. At the same time, the production of ordinary Portland cement (OPC) is associated with high energy consumption and considerable carbon dioxide emissions. Against the background of green building materials and low-carbon engineering, the partial replacement of conventional cementitious materials with industrial solid wastes has become an important route for resource utilization [5,6,7,8]. Converting industrial solid wastes into engineering materials with cementing and stabilization functions may therefore contribute to waste reduction, resource recovery, and the partial replacement of conventional cementitious materials [9,10,11,12].

Most previous studies on industrial-solid-waste-based stabilization materials have focused on the synergistic use of multiple solid wastes. By exploiting compositional complementarity among different wastes, such systems can improve the reaction environment and, in some cases, enhance strength and structural stability [13,14,15,16]. Previous studies have shown that FA, CG, GGBS, and other industrial by-products can be blended appropriately to prepare stabilization materials with acceptable mechanical properties and durability [17,18,19,20]. For this reason, multi-source utilization has received considerable attention in the field of industrial solid waste valorization.

Nevertheless, multi-source blending places relatively high demands on raw-material type, supply stability, and proportion control. Different industrial solid wastes are usually generated in specific industrial sectors and supplied regionally: CG mainly originates from coal mining and washing, FA from coal-fired power generation, and GGBS from iron and steel production [21,22,23]. The production sites of these wastes may be far apart, and the cross-regional transportation and centralized coordination of multiple wastes can increase logistics costs, storage demands, and implementation complexity [24,25]. In this context, utilizing individual industrial solid wastes near their production sites may reduce cross-regional coordination and better suit engineering scenarios with localized material supply. Clarifying the independent performance of different individual industrial solid waste systems is therefore important for selecting regional waste resources and evaluating their engineering potential.

Compared to multi-source systems, systems incorporating one industrial solid waste type allow the independent performance of each waste to be evaluated without the direct influence of interactions among multiple wastes. Their performance may be influenced by the chemical composition, mineralogy, particle characteristics, and potential reactivity of the incorporated waste [26,27,28,29,30]. Previous studies have examined the stabilization performance of FA, CG, and GGBS separately, but direct comparison remains difficult because the matrix materials, alkali activation conditions, waste dosages, curing regimes, and evaluation indices differ substantially among studies [31,32,33,34]. As a result, the strength response and microstructural characteristics of different individual industrial solid waste systems under identical experimental conditions remain insufficiently understood. In addition, the influence of waste dosage and curing age may differ from one material system to another, and the appropriate dosage and strength-development behavior of each system still need to be clarified under unified testing conditions.

To address this gap, this study established a controlled comparison of FA-, CG-, and GGBS-based individual industrial solid waste systems under identical matrix, activator, curing, and testing conditions. Low-liquid-limit clay was used as the matrix material, and the three industrial solid wastes were independently investigated at waste dosages of 10%, 15%, and 20% and curing ages of 3, 7, and 28 days. Unconfined compressive strength (UCS) tests were conducted to evaluate the effects of waste type, dosage, and curing age on strength development. Representative specimens were further characterized using X-ray diffraction (XRD) and scanning electron microscopy (SEM) to compare their crystalline phase assemblages and microstructural features. The combined UCS, XRD, and SEM analyses were used to examine the differences in dosage- and age-dependent strength development and to evaluate whether the observed strength differences could be explained by the identified crystalline phases and qualitative microstructural features. This controlled comparison therefore provides a basis for understanding the independent performance of FA, CG, and GGBS in clay stabilization under the investigated conditions. 

## 2. Materials and Methods

### 2.1. Raw Materials

FA, CG, and GGBS were used as representative individual industrial solid wastes. FA and GGBS were obtained from Xitie Mountain Mineral Processing Plant in Haixi Prefecture, Qinghai Province, whereas CG was obtained from the Yuka Coal Mine in Haixi Prefecture, Qinghai Province, China. The CG was water-washed and used without calcination or thermal activation.

**Figure 1:**
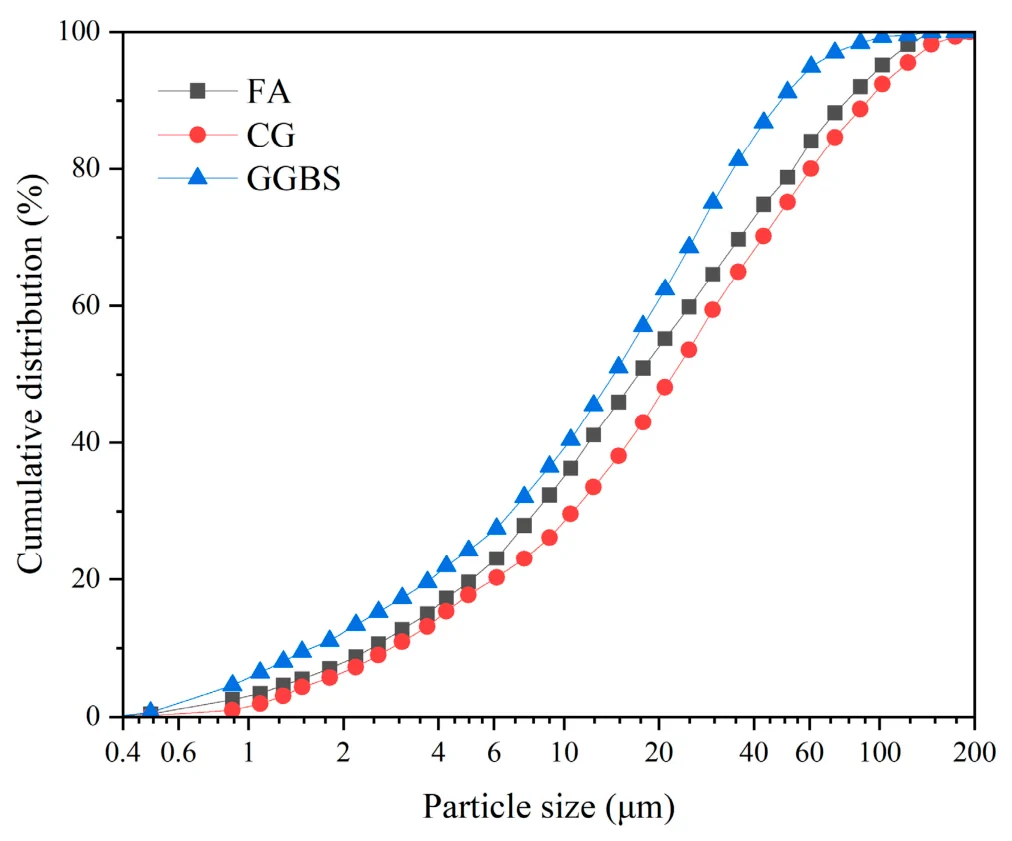
Particle-size distribution curves of FA, CG, and GGBS.

Before use, FA, CG, and GGBS were oven-dried at 105 °C to constant mass, mechanically crushed into powder, and passed through a 2 mm sieve. Their particle-size distribution curves are shown in Figure 1, and their chemical compositions are presented in Table 1.

OPC was used as the conventional cementitious-material reference. NS with a modulus of 2.85 ± 0.10 was used as the activator. It contained approximately 20–23 wt.% Na_2_O and 55–64 wt.% SiO_2_. The NS dosage was calculated based on the mass of the commercial powdered product relative to the dry mass of the clay matrix.

The matrix material was low-liquid-limit clay collected from Qinghai, China. Before testing, the clay was oven-dried, ground, and passed through a 2 mm sieve, after which it was sealed and stored until use. Its primary mineral composition and basic physical properties are presented in Table 2 and Table 3.

### 2.2. Experimental Design

This study aimed to compare the stabilization performance and microstructural characteristics of different individual industrial solid waste systems under consistent experimental conditions. Accordingly, three types of experimental systems were established: an ordinary Portland cement reference system, a sodium silicate baseline system, and individual industrial solid waste systems. The mixture proportions are presented in Table 4.

The ordinary Portland cement system was prepared with a cement dosage of 4% to provide a reference for conventional cementitious materials. The sodium silicate baseline system included five sodium silicate dosages of 2%, 3%, 4%, 5%, and 6%, which were used as a preliminary screening to select a common sodium–silicate treatment condition for the subsequent comparative tests. Because this screening was based only on the 3-day UCS results, the selected dosage was not considered a universal optimum for long-term strength development.

The individual industrial solid waste systems consisted of fly ash, coal gangue, and ground granulated blast-furnace slag. These materials were added separately to the fixed clay matrix rather than replace part of the clay. Each system was treated with 2% sodium silicate and prepared with three waste dosages of 10%, 15%, and 20% to compare the effects of waste type and dosage on strength development.

All experimental groups were cured for 3, 7, and 28 days. UCS tests were conducted to evaluate the strength-development characteristics of the different systems. On this basis, representative specimens cured for 28 days were selected for X-ray diffraction (XRD) and scanning electron microscopy (SEM) analyses to examine the relationship between the strength response, crystalline mineral composition, and microstructural characteristics of the tested systems.

For all mixtures, the clay mass and mixing-water mass were fixed at 180 g and 36 g, respectively. The dosages of OPC, sodium silicate, and industrial solid wastes were calculated as percentages of the dry mass of the clay matrix. FA, CG, and GGBS were added to the fixed clay matrix and did not replace any portion of the clay. Consequently, the total solid mass varied with the industrial solid waste dosage.

### 2.3. Specimen Preparation, Curing, and Characterization

Each specimen was prepared using 180 g of matrix material and 36 g of mixing water. For the sodium silicate-containing systems, the designed amount of sodium silicate was first dissolved in the mixing water to prepare a homogeneous sodium silicate solution. The matrix material was then dry-mixed with cement or the corresponding industrial solid waste to ensure sufficient homogenization of the solid components. Subsequently, the sodium silicate solution was added, and mixing was continued until a uniform mixture was obtained. The ordinary Portland cement reference specimens were prepared using the same mixing procedure, but without the addition of sodium silicate.

The specimens were prepared using detachable cylindrical molds with an inner diameter of 39.1 mm and a height of 80 mm. Before molding, the inner surfaces of the molds and the contact surfaces of the mold components were coated with a thin layer of petroleum jelly, and release paper was placed at the bottom of each mold. The mixed material for each specimen was divided into two equal portions and compacted in two layers. Each portion was slowly introduced into the mold through a funnel and compacted using a manual hydraulic press. The first layer was compressed to the first reference mark and held for 30 s. After pressure release, the surface of the first layer was lightly roughened before the second portion was added. The second layer was then compacted to the final reference mark and held for another 30 s. After demolding, the specimen edges and loading surfaces were trimmed to obtain regular cylindrical specimens. The actual dry density of each specimen was calculated from the dry mass of the mixture and the fixed mold volume.

After demolding, each specimen was completely wrapped with plastic film, placed in a sealed bag, and cured in a standard curing chamber at 20 ± 2 °C and a relative humidity of not less than 95%.

UCS tests were conducted in accordance with the unconfined compression test method specified in the Standard for Soil Test Methods (GB/T 50123-2019) [35] using a computer-controlled electro-hydraulic servo universal testing machine manufactured (Jinan Chenda Testing Machine Manufacturing Co., Ltd., Jinan, China). The loading rate was set to 1 mm/min. Before testing, both ends of each specimen were trimmed to obtain relatively flat loading surfaces. Three parallel specimens were tested for each mixture, and the mean UCS and standard deviation were calculated. The strength results are presented with error bars in the corresponding figures. For the 28-day UCS results, one-way analysis of variance (ANOVA) followed by Tukey’s honestly significant difference (HSD) test was used to evaluate the effect of waste dosage at a significance level of 0.05.

UCS tests were conducted after curing for 3, 7, and 28 days to evaluate the mechanical performance of the different systems. Representative specimens cured for 28 days were selected for X-ray diffraction (XRD) and scanning electron microscopy (SEM) analyses to examine the differences in crystalline mineral composition and microstructural features among the tested systems.

Before the XRD tests, the specimens were crushed, ground, and prepared as powder samples. The samples were immersed in anhydrous ethanol for 3 days to terminate further hydration, dried at 60 °C to constant mass, and then ground to pass through a 0.075 mm sieve. XRD analysis was performed using a D8 ADVANCE diffractometer (Bruker AXS SE, Karlsruhe, Germany) with Cu Kα radiation at an operating voltage of 40 kV and a current of 150 mA. The diffraction patterns were collected over a 2θ range of 5–70° with a step size of 0.02° per step.

Before the SEM tests, representative portions were collected from the interior of the specimens, crushed into fragments of approximately 5–10 mm, immersed in anhydrous ethanol for 3 days, and dried at 60 °C to constant mass. The specimens were subsequently surface-trimmed, mounted on sample holders, and gold-coated before observation. SEM images were acquired using a TESCAN MIRA3 scanning electron microscope (TESCAN GROUP, a.s., Brno, Czech Republic) at an accelerating voltage of 10.0 kV with a secondary-electron detector.

Quantitative phase analysis was performed using the whole-pattern fitting (WPF) and Rietveld refinement functions of MDI JADE 9 software. The crystalline phases were identified with reference to the ICDD Powder Diffraction File (PDF) database, and the corresponding PDF card numbers were recorded in the analysis reports. The diffraction data were fitted over the 2θ range of 5–70° using Cu Kα radiation (λ = 1.54059 Å), with a step size of 0.02° per step. A pseudo-Voigt function was used to model the peak profiles, and a fixed-background model was applied. Sample-displacement correction and Kα_2_-related settings were included according to the refinement control file. The refinement was terminated after 24–27 iterations for the analyzed specimens. The fitting quality was evaluated using the R value reported by the JADE software, which was 9.60%, 9.22%, 6.56%, 8.91%, and 6.27% for NS-2, OPC-4, FA-NS-15, CG-NS-15, and GGBS-NS-15, respectively.

The XRD and SEM results were combined to discuss the microstructural characteristics of the different individual industrial solid waste systems and their relationship with the macroscopic strength response.

## 3. Results and Discussion

### 3.1. Unconfined Compressive Strength of the Reference Systems

**Figure 2:**
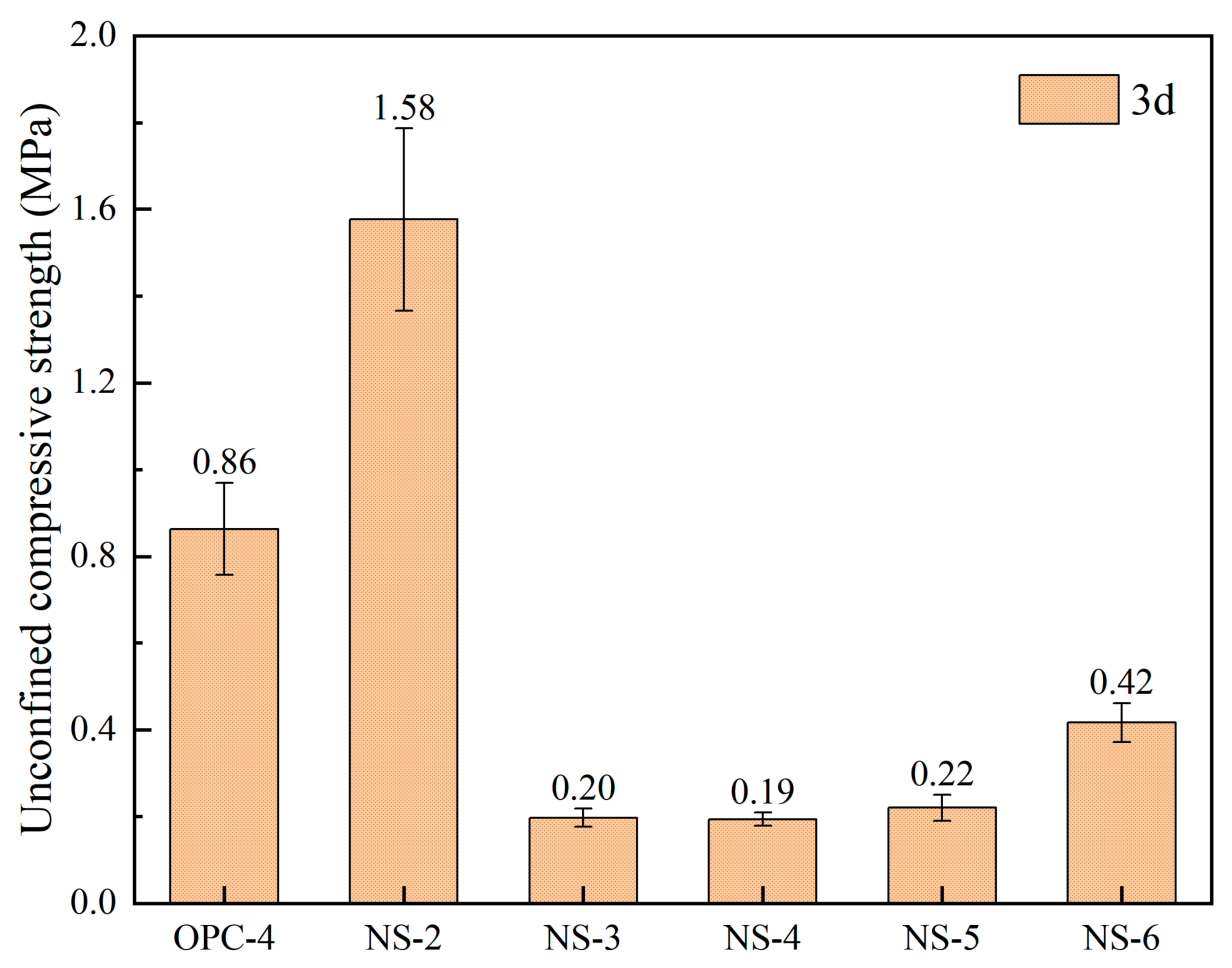
3d unconfined compressive strength of the reference systems.

To select the sodium silicate dosage used in the subsequent individual industrial solid waste tests, the 3-day UCS of specimens prepared with different sodium silicate dosages were compared with that of the OPC-4 reference system (Figure 2).

NS-2 exhibited the highest 3-day UCS, reaching 1.58 MPa, which was 1.84 times that of OPC-4 (0.86 MPa). The 3-day UCS values of NS-3, NS-4, NS-5, and NS-6 were 0.20, 0.19, 0.22, and 0.42 MPa, respectively, all lower than that of NS-2. The strengths of NS-3 to NS-5 were similar, and although the strength of NS-6 increased, it remained well below that of NS-2. These results indicate that, within the investigated dosage range, increasing the sodium silicate dosage did not continuously increase early-age strength.

The relatively high strength of NS-2 indicates that the 2% sodium silicate dosage provided a more suitable treatment condition for the clay matrix under the present experimental conditions. When the sodium silicate dosage increased to 3–5%, the additional sodium silicate did not produce a corresponding increase in strength. This may be related to changes in the balance between particle dispersion, dissolution, and interparticle bonding during the treatment process, which could limit the development of an effective load-bearing structure. The slightly increased strength of NS-6 further indicates that the relationship between sodium silicate dosage and early-age strength was non-monotonic.

Because this comparison was conducted only at 3 d, the results were used solely to select a uniform activator dosage for the subsequent comparative tests of the individual industrial solid waste systems, rather than to evaluate long-term strength development. Therefore, the 2% dosage was adopted as the common sodium silicate dosage under the conditions investigated in this study, rather than being regarded as a universal optimum for all curing ages or material systems. Based on these results, 2% sodium silicate was adopted in the following tests.

### 3.2. Unconfined Compressive Strength of the Individual Industrial Solid Waste Systems

**Figure 3:**
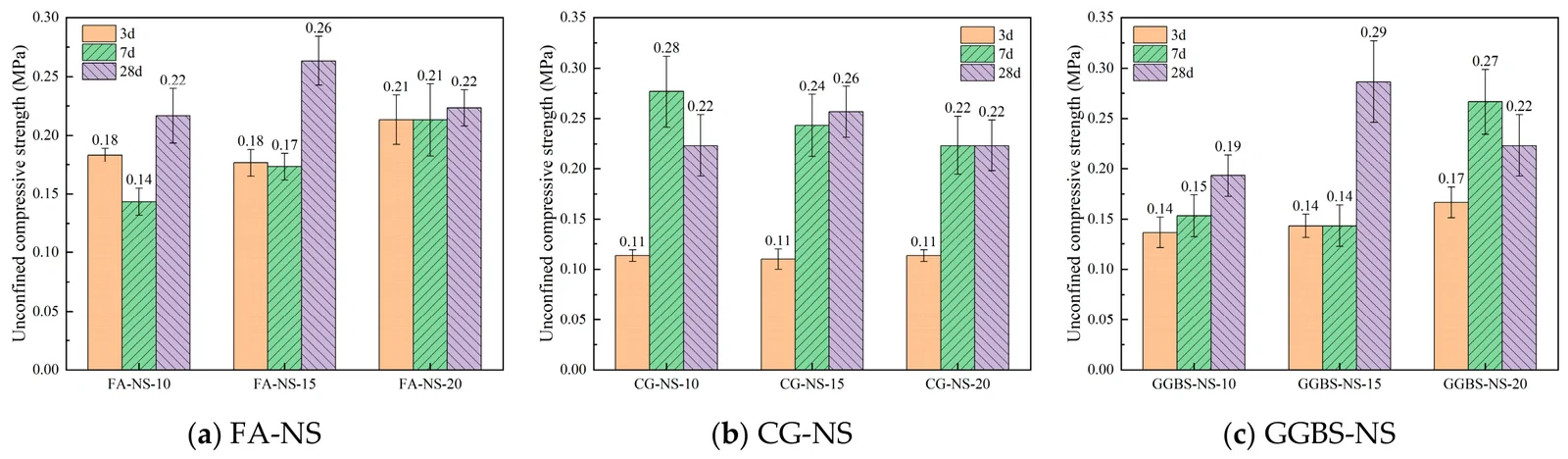
Comparison of the UCS of the individual industrial solid waste systems.

At a fixed sodium silicate dosage of 2%, the UCS results of the three individual industrial solid waste systems are presented in Figure 3. Overall, the three systems exhibited distinct strength-development patterns, and the effect of waste dosage was not consistent across the different waste types.

For comparison, the 3 d UCS of the NS-2 reference system reached 1.58 MPa, whereas that of the industrial-solid-waste systems ranged from 0.11 to 0.21 MPa. This difference suggests that the tested waste-containing systems developed lower early-age strength than the sodium–silicate reference under the adopted conditions, while the primary purpose of these systems was to compare the strength-development behavior of different industrial solid wastes under consistent treatment conditions.

The FA systems showed relatively modest variation with dosage. Their UCS values ranged from 0.18 to 0.21 MPa at 3 d, from 0.14 to 0.21 MPa at 7 d, and from 0.22 to 0.26 MPa at 28 d. At 28 d, FA-NS-10 and FA-NS-20 both reached 0.22 MPa, whereas FA-NS-15 reached 0.26 MPa, the highest measured mean value among the FA systems. However, the difference between FA-NS-15 and the other dosage groups was relatively limited, indicating that the effect of FA dosage was moderate under the conditions investigated.

The CG systems exhibited more pronounced age-dependent differences. All three dosage groups had a 3 d UCS of 0.11 MPa, whereas their 7 d UCS values increased to 0.28, 0.24, and 0.22 MPa, respectively. Their corresponding 28 d UCS values were 0.22, 0.26, and 0.22 MPa. CG-NS-10 reached a local maximum at 7 d and then decreased at 28 d. In contrast, the strength of CG-NS-15 increased continuously from 3 to 28 d, whereas that of CG-NS-20 remained essentially unchanged between 7 and 28 d, with a UCS of 0.22 MPa at both ages. These results indicate that the strength development of the CG systems was sensitive to dosage and curing age, with different dosage groups showing distinct age-dependent trends.

The GGBS systems also exhibited dosage-dependent strength development. The UCS of GGBS-NS-10 gradually increased from 0.14 MPa at 3 d to 0.15 MPa at 7 d and 0.19 MPa at 28 d. GGBS-NS-15 showed limited early-age strength development, with identical UCS values of 0.14 MPa at 3 and 7 d, but its 28 d UCS reached 0.29 MPa, the highest value among the three individual industrial solid waste systems. GGBS-NS-20 reached 0.17 MPa at 3 d and 0.27 MPa at 7 d, followed by a decrease to 0.22 MPa at 28 d. This result indicates that the strength development of GGBS-NS-20 was not strictly monotonic under the tested conditions.

The decreases in UCS from 7 to 28 days observed for CG-NS-10 and GGBS-NS-20 indicate that strength development was not strictly monotonic under the tested conditions. This behavior may be related to changes in the internal structure and the distribution of reaction products during curing. Shrinkage or microcracking may also contribute to the observed strength reduction, although these factors were not directly measured in the present study. The possible effects of carbonation and phase transformation cannot be excluded; however, no direct evidence is available to confirm their contribution. Therefore, the observed decreases are not attributed to a single confirmed mechanism and are interpreted cautiously as non-monotonic strength development.

To further evaluate the effect of waste dosage, one-way analysis of variance (ANOVA) followed by Tukey’s honestly significant difference (HSD) test was performed for the 28 d UCS results of each individual industrial solid waste system at a significance level of 0.05. The dosage effect was not statistically significant for the FA system (F = 4.778, p = 0.058) or the CG system (F = 1.515, p = 0.293). For the GGBS system, the dosage effect was statistically significant (F = 6.811, p = 0.029). Tukey’s HSD test showed that the 15% GGBS dosage produced significantly higher UCS than the 10% dosage, whereas the differences between the 15% and 20% dosages and between the 10% and 20% dosages were not significant. Therefore, although the 15% dosage yielded the highest measured mean 28 d UCS for all three systems, it should be regarded as the dosage associated with the highest measured mean strength within the investigated range, rather than as a statistically confirmed optimum for every system.

Overall, the FA systems showed relatively gradual strength development, the CG systems exhibited more evident intermediate-age variations, and the GGBS system exhibited the most favorable late-age strength at 15% dosage. The highest 28 d UCS values of the FA, CG, and GGBS systems were 0.26, 0.26, and 0.29 MPa, respectively, and all were obtained at 15% dosage. Nevertheless, none of the three systems exhibited a continuous increase in strength with increasing waste dosage. This result indicates that the UCS response of the individual industrial solid waste systems depended not only on dosage but also on the compositional characteristics and potential reactivity of the waste materials. Similar composition- and dosage-dependent strength responses have been reported in industrial-solid-waste-based stabilized soils and alkali-activated systems [11,18]. However, the strength levels and age-dependent trends reported in the literature are not necessarily directly comparable because of differences in raw materials, mixture proportions, activator chemistry, and curing conditions. These findings provide the basis for the mineralogical and microstructural analyses presented in the following sections.

### 3.3. Mineralogical Analysis

#### 3.3.1. XRD Patterns

**Figure 4:**
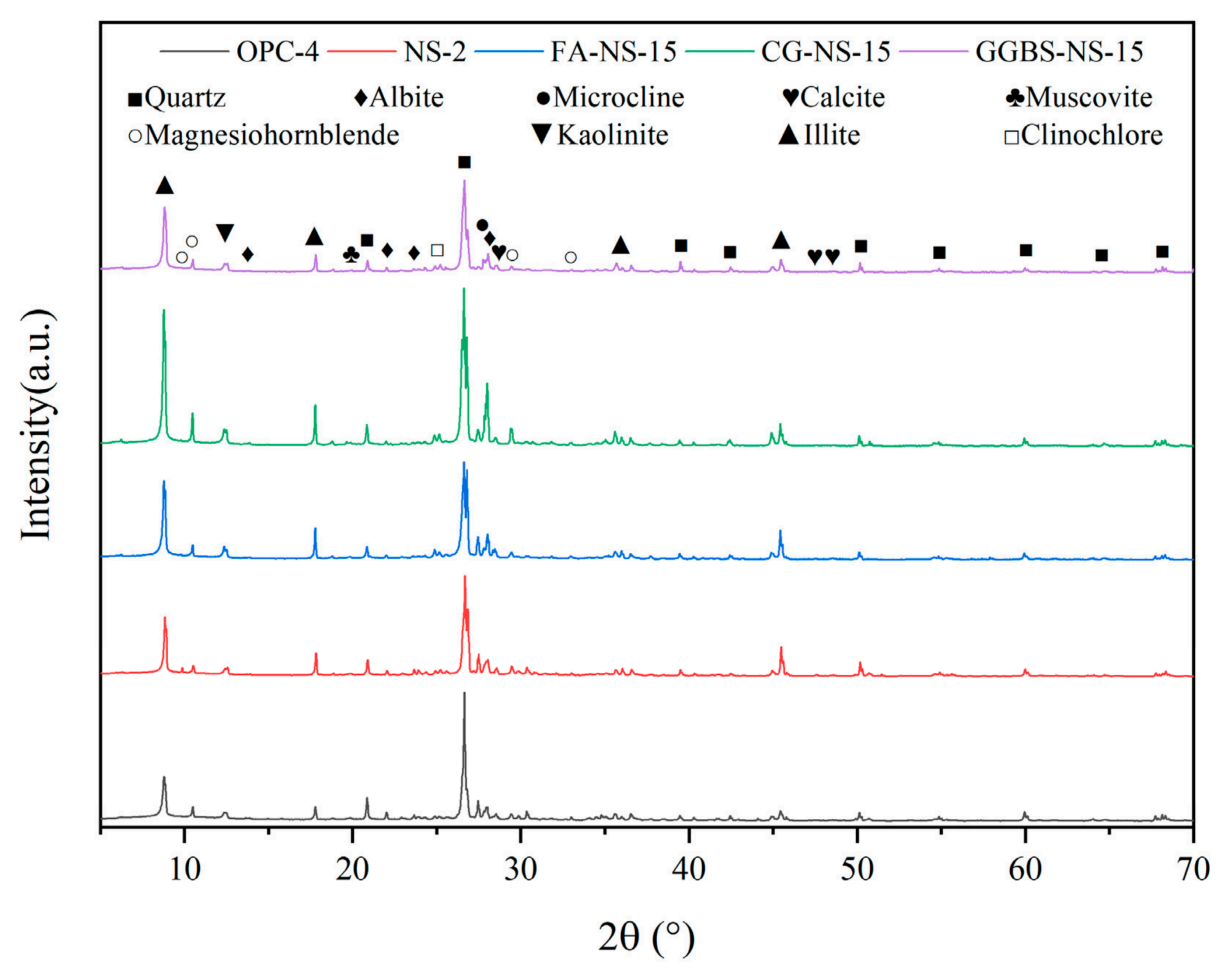
XRD patterns of the different systems after 28 d of curing.

To characterize the mineral assemblages after curing, 28 d specimens were selected for XRD analysis, and the results are shown in Figure 4. FA-NS-15, CG-NS-15, and GGBS-NS-15 represent the specimens with the highest 28 d UCS within the FA, CG, and GGBS series, respectively. For direct comparison, the diffraction patterns were vertically offset.

The XRD patterns of the different systems were broadly similar. The main diffraction peaks corresponded to quartz, illite, albite, microcline, calcite, muscovite, and magnesiohornblende. The quartz peak at approximately 2θ = 26.6° remained evident in all systems, and the characteristic illite peak near 2θ = 8° was also observed, indicating that the main crystalline phases present in the tested mixtures were retained after curing.

The main differences among the systems were reflected in relative peak intensity and local peak shape rather than in peak position, and no obvious new crystalline peaks were detected. In CG-NS-15, several peaks associated with clay minerals remained relatively pronounced, suggesting that part of the original mineral assemblage was retained after the incorporation of coal gangue. FA-NS-15 and GGBS-NS-15 showed peak positions similar to those of OPC-4 and NS-2, although their relative peak intensities differed. This indicates that the type of industrial solid waste mainly influenced the proportions of existing crystalline phases rather than generating a substantially different crystalline phase assemblage.

Although GGBS-NS-15 exhibited the highest 28 d UCS, its XRD pattern did not show a marked increase in newly formed crystalline phases. The strength differences among the systems therefore cannot be explained solely by the formation of new crystalline phases and may also reflect differences in poorly crystalline or amorphous phases, interparticle bonding, and pore-structure development; however, these factors cannot be directly distinguished from the XRD patterns alone. These aspects are examined further using quantitative XRD and SEM observations.

#### 3.3.2. Quantitative XRD Analysis

**Figure 5:**
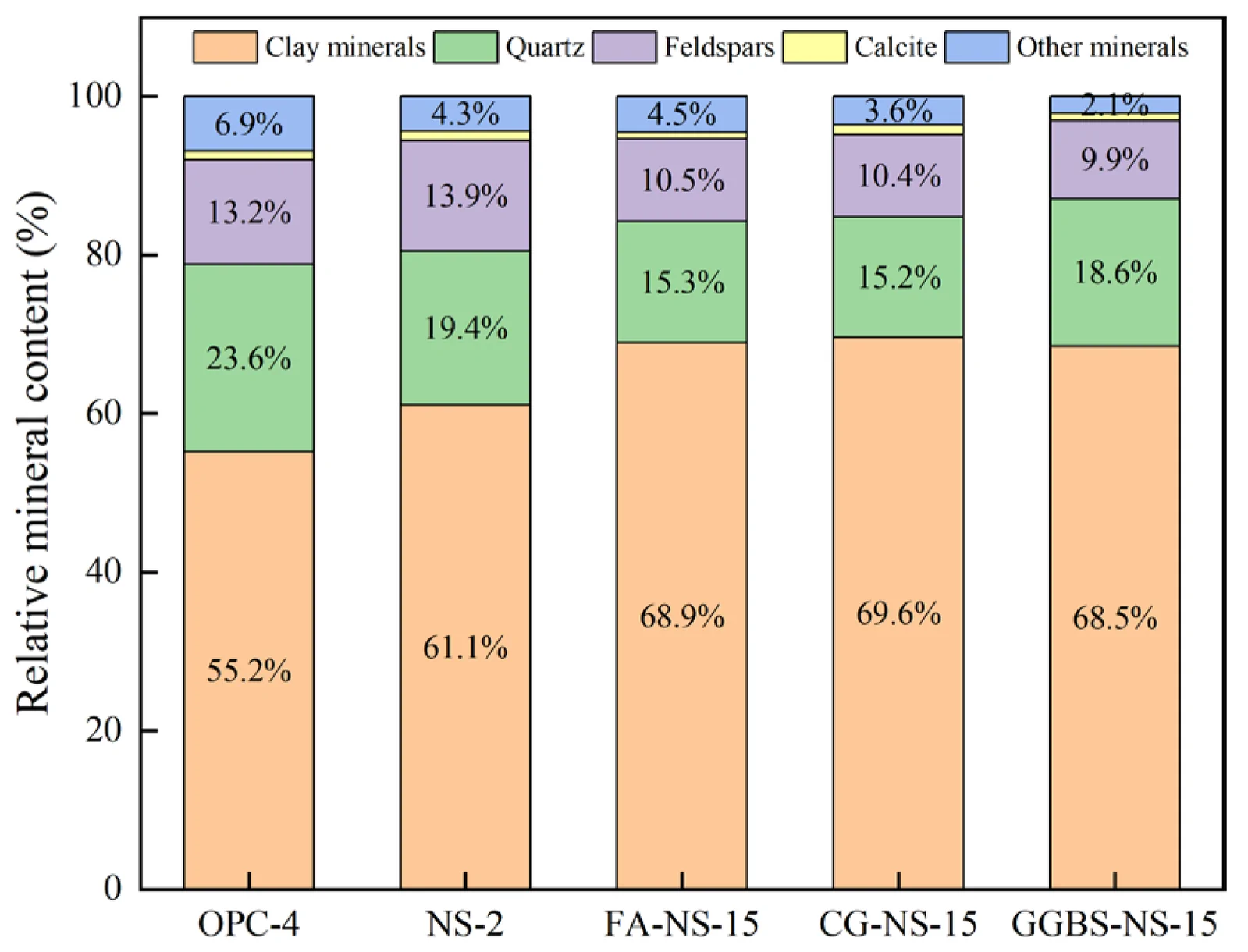
Quantitative crystalline phase composition of the different systems after 28 d of curing.

To further compare the crystalline phase assemblages, quantitative phase analysis was performed using Rietveld full-pattern refinement, and the results are summarized in Figure 5. For clarity, the identified crystalline phases were grouped into five categories: clay minerals (illite, kaolinite, and clinochlore), quartz, feldspars (albite and microcline), calcite, and other minerals (mainly muscovite, magnesiohornblende, and ettringite). The grouped results show broadly similar crystalline assemblages among the different systems. Clay minerals constituted the dominant fraction, ranging from 55.2% to 69.6%, followed by quartz at 15.2–23.6% and feldspars at 9.9–13.9%. Calcite and the other minerals accounted for comparatively small proportions.

Compared to NS-2, OPC-4 showed a lower clay-mineral content (55.2% versus 61.1%), a higher quartz content (23.6% versus 19.4%), and a slightly lower feldspar content (13.2% versus 13.9%). These changes indicate that the introduction of cement altered the relative proportions of the pre-existing crystalline phases. A small amount of ettringite was detected only in OPC-4, indicating limited hydration of the cement component and formation of a minor sulfate-bearing aluminate phase. Because its content was very low, ettringite is more appropriately discussed in the text than plotted as an independent category in the stacked bar chart.

In the three individual industrial solid waste systems, the clay-mineral contents were 68.9%, 69.6%, and 68.5% for FA-NS-15, CG-NS-15, and GGBS-NS-15, respectively, all higher than those of NS-2 and OPC-4. The corresponding quartz contents were 15.3%, 15.2%, and 18.6%, respectively, and were generally lower than those of the two reference systems. FA-NS-15 and CG-NS-15 exhibited similar grouped mineral compositions, whereas GGBS-NS-15 showed a relatively higher quartz content and a slightly lower clay-mineral content. These results indicate that the intrinsic mineralogical characteristics of the different industrial solid wastes affected the relative proportions of crystalline phases in the treated systems.

Notably, GGBS-NS-15 exhibited the highest 28 d UCS, although its crystalline phase composition did not show substantial formation of new crystalline phases. Therefore, the higher strength cannot be explained solely by the relative contents of the identified crystalline phases. Previous studies on alkali-activated fly ash and slag-containing systems have also shown that strength development involves factors beyond the identified crystalline phases [27,30]. Differences in poorly crystalline or amorphous phases may be one possible factor; however, this possibility was not directly verified in the present study.

The Rietveld results quantify only the identified crystalline phases and do not directly measure the amorphous fraction or the overall reaction degree. In addition, no internal standard was used to independently determine the amorphous content. Consequently, the reported phase contents represent normalized relative proportions, and differences among the crystalline phases may partly result from the normalization procedure. The higher apparent clay-mineral contents in the individual industrial solid waste systems therefore do not necessarily indicate an actual increase in the absolute amount of clay minerals.

The different strength-development patterns may be related to differences in the composition and potential reactivity of FA, CG, and GGBS. Similar composition-dependent differences in strength development have been reported for alkali-activated fly ash and slag-containing systems [27,30]. Consistent with these studies, the present results show that the three waste types exhibited different strength-development patterns under the same sodium–silicate treatment. Differences in the reactive components may influence the development of poorly crystalline or amorphous binding phases, which may contribute to interparticle bonding and matrix densification. However, unlike studies with more detailed reaction-product characterization, the present XRD results do not directly quantify these phases or the overall reaction degree. Therefore, the comparison supports a possible relationship between material composition, reaction development, and strength, but does not confirm a specific reaction mechanism in the present systems.

However, the present XRD and SEM results do not allow the specific reaction pathways, reaction degrees, or individual contributions of amorphous products to be quantitatively distinguished. Therefore, the proposed mechanisms are interpreted as composition- and microstructure-based explanations supported by the UCS, XRD, and SEM results, rather than as direct confirmation of a specific pozzolanic or alkali-activation reaction. These interpretations remain tentative because the amorphous fraction and specific reaction products were not directly characterized in the present study.

### 3.4. SEM Observations and Microstructural Analysis

**Figure 6:**
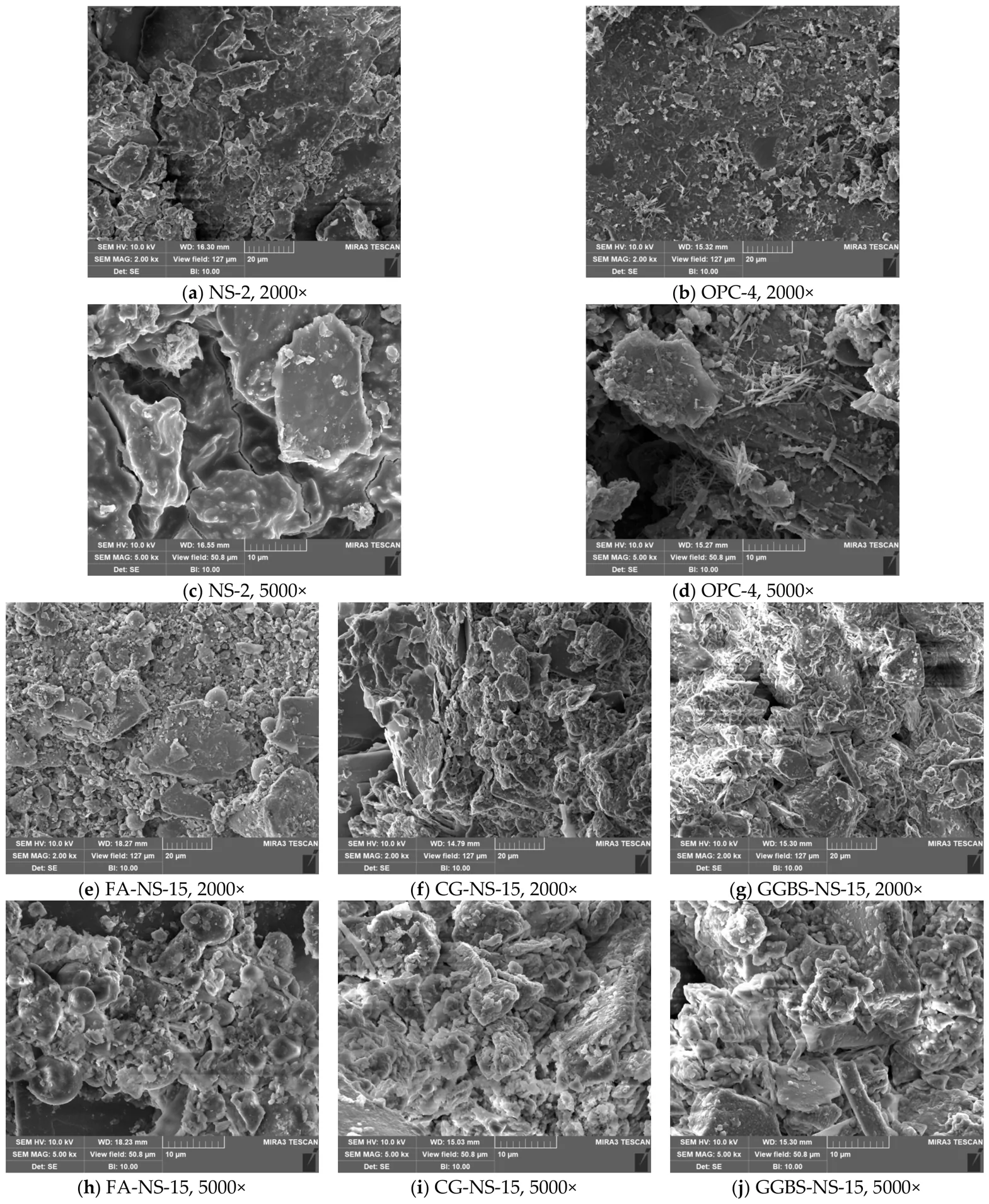
SEM micrographs of the investigated systems after 28 d of curing.

To compare the microstructural characteristics of the different systems, 28 d specimens were examined by SEM, as shown in Figure 6. The main differences among the systems were observed in particle morphology, surface-product coverage, interparticle bonding, and pore distribution. Because the SEM observations were qualitative, the following descriptions are used to compare visible morphological features rather than to provide quantitative measurements of bonding or pore structure.

Figure 6a,c shows the microstructure of NS-2. At low magnification, plate-like and blocky particles are stacked together, and fine products are distributed over part of the particle surfaces. At high magnification, local interparticle bonding can be observed, although distinct particle boundaries and discontinuous regions remain visible. These features suggest that some cementation occurred within NS-2, while the overall structure still retained a pronounced particle-stacking character.

Figure 6b,d shows the microstructure of OPC-4. Compared to NS-2, OPC-4 showed more visible surface deposits and locally filled regions, with fewer clearly open gaps in the observed areas. Needle-like products were observed at high magnification; together with the corresponding XRD result, these features may be associated with a small amount of ettringite, although their morphology alone cannot confirm this phase. Overall, the observed features suggest relatively improved local particle connection in OPC-4 compared with NS-2.

Figure 6e,h shows the microstructure of the FA-NS-15 system. A large number of approximately spherical particles can be observed, together with plate-like and irregular particles. Fine deposits partially covered the particle surfaces, but relatively intact particle boundaries and local pores remained visible. These observations suggest that surface filling and particle connection were locally developed, while discontinuous regions remained visible, which is consistent with the relatively gradual strength development of the FA systems.

Figure 6f,i shows the microstructure of the CG-NS-15 system. Its morphology was dominated by plate-like, blocky, and irregular particles. Particle boundaries remained relatively distinct, and gaps and insufficiently filled regions were visible between particles. Compared with the FA-NS-15 system, the CG-NS-15 system showed more irregular particle features and more visibly discontinuous regions in the observed areas. These qualitative observations are consistent with the different age-dependent strength variations observed in the CG systems, but they do not by themselves establish the cause of those variations.

Figure 6g,j shows the microstructure of the GGBS-NS-15 system. Plate-like and flocculent features were observed on the particle surfaces, and some regions showed closely contacted particles with relatively fewer visible gaps. These features were particularly evident in the observed GGBS-NS-15 areas and are consistent with its relatively high 28 d UCS. However, local pores and incompletely filled regions were still present, indicating that the observed structure remained non-uniform. The plate-like and flocculent features cannot be assigned to specific reaction products solely from the SEM images.

Overall, the three individual industrial solid waste systems exhibited clear differences in particle morphology, surface deposits, and local particle contact. Spherical particles were most prominent in the FA-NS-15 system, whereas the CG-NS-15 system was dominated by plate-like, blocky, and irregular particles. The GGBS-NS-15 system showed more visible surface deposits and local particle contact in the examined areas. Similar qualitative relationships between particle morphology, surface deposits, interparticle contact, and strength development have been reported for industrial-solid-waste-based stabilized and alkali-activated systems [18,19]. The present observations are generally consistent with these reports and provide qualitative complementary evidence for interpreting the differences in strength development. However, they do not quantitatively establish the degree of reaction, the identity of specific reaction products, or the pore-structure parameters of the systems.

### 3.5. Limitations and Engineering Implications

Several limitations should be considered when interpreting the results of this study. Only one clay material and one source of each industrial solid waste were investigated. The experimental program included one sodium–silicate treatment system, three waste dosages of 10%, 15%, and 20%, and curing ages of 3, 7, and 28 d. All specimens were cured at 20 ± 2 °C and a relative humidity of not less than 95% to provide consistent comparison among the tested systems. However, because no additional curing temperatures were investigated, the effects of temperature on the reaction kinetics and strength development of the FA-, CG-, and GGBS-containing systems could not be evaluated or compared. Temperature may affect the dissolution of reactive components and the subsequent formation of binding products, and the sensitivity of these processes may differ among FA, CG, and GGBS. However, this possible temperature dependence was not evaluated in the present study. Therefore, the results are specific to the tested materials and conditions and should not be generalized to all FA, CG, or GGBS systems.

The characterization was mainly based on XRD and SEM. As discussed in Section 3.3.2 and Section 3.4, these techniques do not directly quantify the amorphous fraction, specific chemical bonding, detailed reaction degree, or pore-structure parameters. Complementary analyses, such as internal-standard XRD, SEM coupled with energy-dispersive X-ray spectroscopy (EDS), Fourier transform infrared spectroscopy (FTIR), thermogravimetric analysis (TGA), and Brunauer–Emmett–Teller (BET), would be useful for further verifying the reaction products and microstructural changes. In addition, the environmental safety of the stabilized materials was not evaluated. The potential leaching of heavy metals from the industrial solid wastes and the ability of the treatment to immobilize such contaminants therefore remain uncertain.

From an engineering perspective, the measured 28 d UCS values of 0.22–0.29 MPa are relatively low and cannot by themselves demonstrate compliance with the requirements for structural foundation materials or high-load road-base applications. For engineering context, the current Polish guideline WR-D-61 lists hydraulically stabilized soil with strength class C_0.4/0.5_ as a possible material for an improved subgrade layer for pavement structures covering traffic categories KR1-KR7 (Section 11.34 and Table 11.5) [36]. In this classification, C_0.4/0.5_ corresponds to minimum compressive strengths of 0.4 MPa for cylinders with a slenderness ratio of 2 and 0.5 MPa for cylinders with a slenderness ratio of 1 or cubes. The specimen height-to-diameter ratio in the present study was approximately 2.05, and the measured 28-day UCS values were lower than the 0.4 MPa reference value. However, this comparison is indicative only because the classification in WR-D-61 is not fully equivalent to the testing protocol, specimen preparation, material composition, and curing conditions used in the present study. Therefore, the measured strength levels cannot be directly interpreted as demonstrating compliance with the guideline requirements. Durability assessment is also necessary because stabilized materials may experience repeated environmental actions during service. Wet–dry cycling is needed to evaluate performance under repeated moisture variation, freeze–thaw testing is needed to assess resistance to repeated temperature-induced damage, and chemical-attack testing is needed to examine stability in chemically aggressive environments. Cost, environmental benefits, and long-term field performance were also beyond the scope of this study. Therefore, the present results should be regarded as preliminary comparative evidence for the tested systems, and further application-specific studies are required before their engineering suitability and long-term performance can be determined.

## 4. Conclusions

In this study, the strength development and microstructural characteristics of low-liquid-limit clay incorporating FA, CG, and GGBS were investigated under consistent sodium–silicate treatment and curing conditions. The main conclusions are as follows:

(1) The three industrial solid waste systems exhibited different strength-development patterns. The FA system showed relatively gradual strength development, the CG system showed more evident age-dependent variations, and the GGBS system achieved the highest measured 28 d UCS at a dosage of 15%. The corresponding 28 d UCS values of the FA, CG, and GGBS systems were 0.26, 0.26, and 0.29 MPa, respectively. ANOVA results indicated that the dosage effect was not statistically significant for the FA or CG system, whereas it was significant for the GGBS system. Tukey’s HSD test further showed that the 15% GGBS dosage was significantly higher than the 10% dosage, but its differences from the 20% dosage were not significant.

(2) XRD analysis showed that the tested systems had broadly similar crystalline-phase assemblages, mainly consisting of clay minerals, quartz, and feldspars. The addition of FA, CG, and GGBS mainly changed the relative proportions of the identified crystalline phases, while no abundant new crystalline phases were detected. The higher strength of GGBS-NS-15 was therefore associated with its overall material and structural characteristics rather than with a substantial increase in newly identified crystalline phases.

(3) SEM observations showed visible differences in particle morphology, surface deposits, and local particle contact. Spherical particles were more prominent in the FA system, while plate-like, blocky, and irregular particles were more evident in the CG system. The GGBS system showed more visible surface deposits and local particle contact, consistent with its relatively high 28 d UCS.

Overall, the results demonstrate that waste type and dosage jointly influenced the strength development and microstructural characteristics of the tested systems. The findings provide useful comparative information for evaluating FA, CG, and GGBS as supplementary components in low-strength soil stabilization under the investigated conditions.

## Figures and Tables

**Table 1:** Chemical compositions of FA, CG, and GGBS (wt.%).

Material	SiO_2_	Al_2_O_3_	Fe_2_O_3_	CaO	MgO	SO_3_	TiO_2_
FA	57.96	23.33	4.61	5.03	-	-	1.61
CG	56.43	27.67	5.42	3.83	2.14	-	0.57
GGBS	23.62	13.52	0.52	53.01	7.18	1.74	-

**Table 2:** Mineral composition of the low-liquid-limit clay.

Mineral	Content (wt.%)
Quartz	20.3
Albite	5.9
Microcline	2.7
Calcite	1.8
Muscovite	2.8
Magnesiohornblende	3.3
Kaolinite	1.2
Illite	56.5
Clinochlore	5.3
Portlandite	0.2

**Table 3:** Basic physical properties of the low-liquid-limit clay.

Specific Gravity	Porosity (%)	Liquid Limit (%)	Plastic Limit (%)
2.63	27.74	21.5	13.0

**Table 4:** Experimental groups and mixture proportions.

Group	OPC Dosage (%)	NS Dosage (%)	Solid Waste Type	Solid Waste Dosage (%)
OPC	4	-	-	-
NS	-	2, 3, 4, 5, 6	-	-
FA-NS	-	2	FA	10, 15, 20
CG-NS	-	2	CG	10, 15, 20
GGBS-NS	-	2	GGBS	10, 15, 20

Note: The dosages of all additional components were calculated relative to the dry mass of the clay matrix. FA, CG, and GGBS were added to the clay rather than used to replace part of it.

## Data Availability

The original contributions presented in this study are included in the article. Further inquiries can be directed to the corresponding author.
